# In Vitro Cell Toxicity and Intracellular Uptake of Doxorubicin Exposed as a Solution or Liposomes: Implications for Treatment of Hepatocellular Carcinoma

**DOI:** 10.3390/cells10071717

**Published:** 2021-07-06

**Authors:** Fredrik Kullenberg, Oliver Degerstedt, Carlemi Calitz, Nataša Pavlović, David Balgoma, Johan Gråsjö, Erik Sjögren, Mikael Hedeland, Femke Heindryckx, Hans Lennernäs

**Affiliations:** 1 Department of Pharmaceutical Biosciences, Uppsala University, 75 123 Uppsala, Sweden; fredrik.kullenberg@farmbio.uu.se (F.K.); oliver.degerstedt@farmbio.uu.se (O.D.); johan.grasjo@ilk.uu.se (J.G.); erik.sjogren@farmbio.uu.se (E.S.); 2 Department of Medical Cell Biology, Uppsala University, 75 123 Uppsala, Sweden; C.calitz@amsterdamumc.nl (C.C.); natasa.pavlovic@mcb.uu.se (N.P.); femke.heindryckx@mcb.uu.se (F.H.); 3 Department of Medicinal Chemistry, Uppsala University, 75 123 Uppsala, Sweden; david.balgoma@ilk.uu.se (D.B.); mikael.hedeland@ilk.uu.se (M.H.)

**Keywords:** hepatocellular carcinoma, doxorubicin, liposome, cell model, intracellular concentration, physiologically based pharmacokinetic model

## Abstract

Cytostatic effects of doxorubicin in clinically applied doses are often inadequate and limited by systemic toxicity. The main objective of this in vitro study was to determine the anti-tumoral effect (IC_50_) and intracellular accumulation of free and liposomal doxorubicin (DOX) in four human cancer cell lines (HepG2, Huh7, SNU449 and MCF7). The results of this study showed a correlation between longer DOX exposure time and lower IC_50_ values, which can be attributed to an increased cellular uptake and intracellular exposure of DOX, ultimately leading to cell death. We found that the total intracellular concentrations of DOX were a median value of 230 times higher than the exposure concentrations after exposure to free DOX. The intracellular uptake of DOX from solution was at least 10 times higher than from liposomal formulation. A physiologically based pharmacokinetic model was developed to translate these novel quantitative findings to a clinical context and to simulate clinically relevant drug concentration–time curves. This showed that a liver tumor resembling the liver cancer cell line SNU449, the most resistant cell line in this study, would not reach therapeutic exposure at a standard clinical parenteral dose of doxorubicin (50 mg/m^2^), which is serious limitation for this drug. This study emphasizes the importance of in-vitro to in-vivo translations in the assessment of clinical consequence of experimental findings.

## 1. Introduction

The anthracycline doxorubicin (DOX) is a well-established chemotherapeutic drug, commonly used to treat solid tumors and hematologic malignancies. While DOX treatment has shown great potential in slowing down disease progression over the past decades, clinically used doses often show insufficient anti-tumor efficacy, whereas higher doses frequently result in systemic toxicity in patients. Therefore, there is a pressing need to develop drug delivery strategies with increased DOX-induced anti-tumor potency, while reducing off-target toxicity to noncancerous tissues [1].

Doxorubicin and its main metabolite doxorubicinol (DOXol) induce cytotoxicity by multiple mechanisms of action, including triggering apoptosis and cell cycle arrest by DNA intercalation and inhibition of DNA repair mechanisms, as well as generating reactive oxygen species, thereby causing further DNA damage [2]. A major factor that determines the anti-tumoral potency of chemotherapeutics is the intracellular drug exposure, as well as the accumulation of the drug and its active metabolites in its target. Passive membrane diffusion is one of the key-mechanisms for DOX cellular transport, for which the amphiphilic nature of DOX and a Log D_7.4_ of 2.4 are key properties. Other carrier-mediated processes, such as solute carrier transporter SLCO1A2 and SLC22A16, and ATP-binding cassette transporter-mediated efflux, are also involved in the net influx into the cells [3,4,5,6]. Another important factor in the cellular uptake and cytotoxicity of chemotherapeutics is the pH gradient [7,8,9]. Doxorubicin has a basic pKa of 8.2 and has been reported previously to become sequestered in the acidic conditions in late stage endosomes and lysosomes [10]. The intracellular DOX exposure might be affected by concomitant dosing of a proton pump inhibitor (PPI), such as lansoprazole and pantoprazole, as it has been shown to raise pH in endosomes [11,12].

Several modified-release parenteral drug delivery systems with DOX have been developed in an effort to prolong local tumor drug exposure and reduce off-target toxicity [13,14]. This includes therapeutic nanoparticles (TNPs) such as Doxil^®^, a pegylated liposomal doxorubicin (DOX_PL_) formulation for intravenous administration, where DOX is encapsulated in liposomes covered with a layer of polyethylene glycol coating. The intra-particular drug load of DOX is more than 90% in DOX_PL_ (Doxil^®^) with a size range of 80–100 nm in diameter [15]. The proposed mechanism for TNP uptake and non-specific targeting in solid tumors has been attributed to the enhanced permeability and retention (EPR) effect [16]. Rapid tumor proliferation is known to induce the formation of dysfunctional blood vessels with a defective and leaky endothelium, which allows for particles smaller than 2000 nm to passively enter the tumor tissue. This, along with the impaired lymphatic drainage, commonly results in EPR. However, while TNPs have been reported to exhibit improved anti-tumor effects in a variety of animal models, the translational and clinical relevance of the EPR effect of TNPs has been challenged [17,18,19]. Furthermore, while DOX_PL_ has prolonged the total plasma exposure of DOX and improved safety properties compared to formulations with free DOX (DOX_S_) in patients, it has failed to enhance the therapeutic anticancer efficacy [13,20,21].

Hepatocellular carcinoma (HCC), one of the most common and most deadly malignancies worldwide, is commonly treated with DOX at the intermediate disease stage [1]. In an effort to reduce the toxic side-effects of intravenous DOX therapy in HCC patients, several locoregional treatment strategies have been developed, including transarterial chemo-embolization (TACE) [1,20,22]. This technique uses high-precision image guidance to locally dose the drug delivery system through the tumor’s artery, thereby creating a high local concentration in the tumor tissue, while reducing the systemic exposure and subsequent side effects [14,23,24]. Recently, there has been an increasing interest in combining TACE with therapeutic nanoparticles (TNPs) [25]. However, in order to develop safe and efficient strategies to reach intracellular targets, more insight is needed in regard to tumor cell net uptake of chemotherapeutics and their metabolites, as well as the cytotoxicity and intracellular exposure by chemotherapeutics as free drugs, or different parenteral formulations.

In this study, we aimed to compare the anti-tumoral effect of DOX and DOX_PL_ in vitro, in an effort to fill the existing literature gap in the field and provide more insight into the anti-tumoral potency of these chemotherapeutics. To do this, three primary liver cancer (PLC) cell lines (HepG2, Huh7 and SNU449) and one breast cancer cell line (MCF7) were exposed to a wide concentration range of DOX_S_ and DOX_PL,_ in different exposure times, following which cell viability and cellular uptake of DOX were assessed. Furthermore, in order to improve the understanding of DOX delivery into target tumor cells, the intracellular accumulation of DOX and its active metabolite DOXol was compared, and assessed in relation to its anti-tumor effect in vitro. In addition, PBPK modeling was used to propose how intracellular uptake and cytotoxicity of doxorubicin measured in vitro might be extrapolated to the clinical situation [26,27]. Finally, we investigated how lansoprazole affects the anti-tumor effect of DOX.

## 2. Materials and Methods

### 2.1. Chemicals

Resazurin sodium salt, penicillin-streptomycin amphotericin cocktail, lansoprazole, acetonitrile, ammonium formate, formic acid and phosphate buffered saline (PBS, pH 7.4) tablets were purchased from Sigma-Aldrich (Taufkirchen, Germany). Methanol was purchased from Merck Millipore (Darmstadt, Germany) and all solvents were HPLC grade or higher. All water used in the study was of ultra-pure grade (i.e., Milli-Q^®^). High-glucose Dulbecco modified eagle medium with GlutaMAX™ (DMEM), Roswell Park Memorial Institute with GlutaMAX™ 1640 (RPMI), fetal bovine serum (FBS), and trypsin-EDTA were purchased from Gibco. Doxil^®^ (liposomal doxorubicin, marketed as Caelyx^®^ in Europe) was purchased from Apoteket AB (Solna, Sweden). Doxorubicin (DOX) hydrochloride was purchased from Toronto Research Chemicals, Canada. Doxorubicinol (DOXol) trifluoroacetate as well as the internal standards [^13^C, ^2^H_3_]-DOX trifluoroacetate (DOX IS) and [^13^C, ^2^H_3_]-DOXol trifluoroacetate (DOXol IS) were purchased from Alsachim (Illkirch Graffenstaden, France). Stock solutions (100 mM) for the cell viability experiments were prepared by dissolving DOX hydrochloride in DMSO (Sigma-Aldrich, Taufkirchen, Germany). Lansoprazole stock solution (67.7 mM) was prepared by dissolving lansoprazole in DMSO.

### 2.2. Overall Study Design and Investigational Drugs

In this study, the intracellular uptake and antitumor effect of DOX was investigated following exposure of two different formulations in four human cancer lines (described in detail below). One study drug was DOX as a solution (DOX_S_) and the second was pegylated liposomal DOX (DOX_PL_; Doxil^®^). The micro-PK and pharmacodynamics (PD) were monitored during 24-, 48- and 72-h exposure at DOX concentrations of 0.001–1000 µM and 0.1–1000 µM for DOX_S_ and DOX_PL_, respectively. The effect of lansoprazole (500 µM) on the two study formulations DOX_S_ and DOX_PL_ was also investigated.

### 2.3. Cell Culture and Culture Conditions

The three PLC cell lines (HepG2, ATCC^®^ HB-8065™, SNU449 ATCC^®^ CRL-2234™, Huh7, kind gift from Dilruba Ahmed, Karolinska Institute, Solna, Sweden) and the human breast cancer cell line MCF7 (kind gift from Johan Kreuger, Uppsala University, Uppsala, Sweden) were cultured at 37 °C with 5% CO_2_ and 95% humidity within a CO_2_ incubator. The HepG2, Huh7 and MCF7 cell lines were routinely cultured in DMEM supplemented with 1% antibiotic antimycotic solution and 10% FBS (cell culture media + FBS: CCM_Fed_). The SNU449 cell line was cultured RPMI medium supplemented with 1% antibiotic antimycotic solution and 10% FBS (cell culture media + FBS: CCM_Fed_). Standard culture medium without supplemented FBS was used during starvation (CCM_SM_). Misidentification of all cell lines was checked at the Register of Misidentified Cell Lines, and none of the chosen cell lines were on the list [28]. Extracted DNA from all four cell lines were sent to Eurofins Genomics (Ebersberg, Germany) for cell line authentication using DNA and short tandem repeat-profiles. Authentication confirmed the correct identity of each cell line and each cell line tested negative for mycoplasma contamination. The choice of cell lines was based on earlier validation of their IC_50_ values for DOX during normal and hypoxic conditions. SNU449 was selected as it is one of the most drug-resistant commercially available cell lines, which is not genetically engineered to be chemo-resistant [29]. A breast cancer cell line, MCF7, was also included in the study to reflect the current clinical usage of Doxil^®^, which is treatment of breast cancer in Europe and Canada.

### 2.4. Drug Treatment Schedule

Cells were plated at a seeding density of 1 × 10^4^ cells/well in 200 µL CCM_Fed_ onto clear black flat-bottom 96-well plates (Costar, USA), with the outermost wells filled with PBS to minimize the edge effect [30]. The cells were allowed to attach overnight. Prior to treatment, CCM_Fed_ was removed, and the cells washed with 200 µL PBS. To allow synchronization of the cell cycle, 200 µL CCM_SM_ was added to each well containing cells for 2 h prior treatment [30]. After cell cycle stabilization, cells were pretreated with 500 µM lansoprazole in CCM_Fed_ for two hours (when applicable), followed by treatment with DOX_S_ or DOX_PL_. For DOX_S_, the DOX stock solution in DMSO was used to create a range of 0.001–1000 µM in CCM_Fed_, and for DOX_PL_ the product Doxil^®^ was used to create a range of 0.1 to 1000 µM in CCM_Fed_. Cells were treated with 200 µL/well of the respective DOX_S_ and DOX_PL_ preparations. Toxicity threshold values of DMSO were also evaluated in separate viability assays for each cell line. To determine the inert range of DMSO concentrations, the maximum dose of DMSO administered during DOX treatment (1% DMSO at 1000 µM DOX solution) times ten was set as the highest concentration. DMSO dilutions in CCM_Fed_ in a concentration range of 0.01–10% (*v*/*v*) was administered to cells 24, 48 and 72 h.

### 2.5. Resazurin-Based Cell Viability Assay

Cell viability was evaluated after drug treatment for 24, 48 or 72 h by a resazurin reduction assay, as previously described [31]. A 1% solution of resazurin sodium salt (Sigma-Aldrich) was prepared according to manufacturer’s recommendation and diluted 1/80 in CCM_SM_. Post treatment, culture medium was removed and the cells washed once with PBS. A volume of 150 µL resazurin solution was added and cells were incubated overnight. The relative fluorescence intensity was measured at excitation and emission wavelengths of 560/5 and 590/5 nm, respectively, on a Tecan Safire II plate reader. Six technical replicates were set up for each experimental group, with three biological replicates for all treatments, except for the pretreatment with lansoprazole where there was only one biological replicate.

### 2.6. Cell Lysate Preparation and Intracellular Determination of DOX and Major Metabolite DOXol

The cells were treated with concentrations of DOX_S_ or DOX_PL_ corresponding to their calculated IC_50_ values, based on the pooled results of the cell viability assays in this study. The maximum concentration used for these experiments was chosen to be 200 µM. Cells were seeded at a seeding density of 4 × 10^6^ cells per T75 flask (75 cm^2^, 60 mL), and allowed to attach overnight. Prior to treatment, CCM_Fed_ was removed, and the cells washed with PBS. To allow synchronization of the cell cycle, 15 mL CCM_SM_ was added to each flask containing cells 2 h prior to treatment. Cells were treated for 24, 48 and 72 h, respectively. Following drug treatment, culture medium was removed and kept on ice. Cells were washed twice with 5 mL PBS, which was collected and kept on ice. A volume of 3 mL trypsin-EDTA was added to each flask, after which they were incubated for 4 min. The resulting cell suspension was collected and diluted with 3 mL CCM_Fed_, and the cells counted using a TC20™ Automated cell counter and counting slides (Bio Rad). Using the histogram/gating option of the automated cell counter the average diameter of the cells was determined. The cell suspension was subsequently centrifuged at 140 g for 5 min, the supernatant removed, stored on ice, and the pellet was resuspended in 1000 µL ice-cold MilliQ water. Samples were lysed by two freeze–thaw cycles, consisting of snap freezing of samples in liquid nitrogen followed by thawing at 37 °C in a water bath for 10 min. After the final thaw cycle, samples were sonicated on ice for 30 s and stored at −20 °C until analysis.

### 2.7. Assay for Quantification of Intracellular Concentrations of DOX and Its Major and Active Metabolite Doxorubicinol (DOXol)

An ACQUITY UPLC I-Class system coupled to a single-quadrupole QDa mass detector (Waters Corporation, Milford, MA) was used to quantify intracellular concentrations of both DOX and its main metabolite DOXol. The mobile phases consisted of (A) 5.0 mM ammonium formate in water:acetonitrile (95:5) with 0.1% formic acid at pH 3.01 ± 0.07 and (B) acetonitrile. The analytes DOX and DOXol were chromatographically separated under the following LC conditions. The gradient used was: initially 5% (B), then a linear increase over 0.00–3.00 min of 5–30% (B), followed by 3.00–3.25 min linear increase to 85% (B), and then held at 85% (B) 3.25–4.25 min. Then linear decrease 4.25–4.50 min of 85–5% (B), and finally 5% (B) maintained 4.50–6.00 min. The total run time was 6 min, the flow rate was 500 μL/min, the sample injection volume was 10 μL, and the sample manager temperature was 10 °C. The column was a C18 column (ACQUITY UPLC BEH C18, 2.1 × 50 mm, particle size 1.7 μm, Waters Corporation) kept at 60 °C. In the single quadrupole QDa detector, the positive electrospray capillary voltage was set at 0.80 kV and probe and source temperatures were 600 °C and 120 °C, respectively. The quantification was performed in single-ion recording mode. The mass detection channels were set to *m*/*z* 544 (DOX), 546 (DOXol), 548 (DOX IS) and 550 (DOXol IS), each with a cone voltage of 8 V. The sampling frequency was 10 Hz.

Stock solutions of analytes and isotopically labelled internal standards were prepared in methanol (1 mg/mL). From these, working standards containing DOX and DOXol were diluted in methanol (0.25–1000 µM) and stored in amber vials at −20 °C. For quantification of DOX_PL_-treated samples, the commercial formulation (2 mg/mL) was diluted to working standards in methanol (5–1000 µM) and stored as mentioned above. All samples were prepared in 96-well 1.00 mL round collection plates from Waters^®^. To construct calibration curves in sample relevant matrices, untreated control sample replicates were pooled and spiked (100 µL) with 25 µL of the appropriate working standard solution (0.25–1000 µM). Chilled (−20 °C) acetonitrile (375 µL) containing the isotopically labelled internal standards at matrix specific amounts (0.3–3 µM) were then added to precipitate cellular proteins. Samples were mixed (by pipetting up and down) before storage overnight at −20 °C. The following day, samples were brought to room temperature before mixing and subsequent centrifugation at 2200 rpm for 3 min at 4 °C. Portions of the supernatants (100 µL) were transferred to a new 96-well collection plate and dried under a gentle stream of nitrogen in a water bath (≈25 °C) using a 3D-printed manifold (U-PRINT) for at least 10 min. The residuals were dissolved in matrix appropriate volumes of mobile phase A (50 to 500 µL) and then injected into the instrument. Linear calibration curves (R^2^ > 0.995) for both DOX and DOXol were constructed between 0.05 and 50 µM for the intracellular and washing matrices and between 1 and 200 µM for the exposure media matrix. A volume of 25 µL of methanol was added to cell portion samples (100 µL) of unknown concentration and they were subsequently treated exactly as the calibration samples. Matrix effects and extraction recoveries were evaluated according to Matuszewski et al. in 2003 [32]. The data were processed using a linear curve fit (weighting factor of 1/x) of the peak area ratio (analyte: internal standard) as a function of the analyte concentration. For both DOX and DOXol, the lowest limit of quantification (LLOQ) in matrix was 50 nM, which corresponds to the lowest point in the linear calibration curves. All the collected data were processed using TargetLynx as part of MassLynx V4.1 (Waters Corporation, Milford, MA, USA).

### 2.8. Data Analysis and Statistics

Cell viability (*V*), defined as the percentage of fluorescence value of treated cells compared to fluorescence value of untreated cells, according to Equation (1) [29]:

(1) $$V=\frac{I_{exp}-{\bar{I}}_{blank}}{{\bar{I}}_{cont}-{\bar{I}}_{blank}}$$

where $I_{exp}$ is the measured fluorescence, ${\bar{I}}_{blank}$ is the average fluorescence of a blank wells containing only the CCM_SM+R_ without any cells in the plate and ${\bar{I}}_{cont}$ is the average of control wells containing cells in CCM_SM+R_.

To accurately calculate viability values and the IC_50_ values, Equation (2), referred to as a four-parameter logistic model equation, was fitted to the experimental data:

(2) $$V\left(x\right)=\frac{\Delta V}{1+{\left(\frac{x}{x_{50}}\right)}^{\gamma }}+V_{\infty }$$

where *x* is the concentration of DOX_S_ or DOX_PL_, Δ*V* the viability at no DOX_S_ or DOX_PL_ exposure, i.e., corresponding to 100% viability, *x*_50_ the concentration of 50% viability, and γ a parameter connected to the slope of the sigmoidal characteristic of the logistic equation. In the fitting, $V_{\infty }$ is the viability at infinite concentration of DOX_S_ or DOX_PL_ and was assumed to be equal to zero and consequently not fitted.

In principle, fitting of Equation (2) also gives *x*_50_. However, the estimation of *x*_50_ using only Equation (2) was found to be highly dependent on the other parameters, and at viability values far from *x*_50_.

The value of *x*_50_ was estimated as the linear interpolation (Equation (3)) between the *x* values corresponding to the *V* value closest above (*V_H_*) and closest below (*V_L_*) the *V*_50_ = Δ*V*/2 value, i.e., half of the 100% viability value:

(3) $$x_{50}=\left(V_{50}-V_{L}\right)\frac{x_{H}-x_{L}}{V_{H}-V_{L}}+x_{L}$$

In any experiment in which the cell viability did not reach less than *V*_50_, determination of *x*_50_ could not be performed, and it was therefore excluded from the analysis. If more than one such experiment was detected for any replicate of a specific treatment condition, the entire treatment condition was excluded from further statistical study. The average and standard deviation of the *x*_50_ values of the different replicate experiments were calculated, and these values are here called IC_50_.

To calculate the IC_10_ values for the cells treated with DMSO, Equation (3) was used and adjusted so *V_H_* and *V_L_* were the viability values closest above and closest below the *V*_90_ = Δ*V* × 0.9 value, i.e., 90% the Δ*V* value. From that equation, the standard error of *IC*_10_, here denoted $s_{IC10}$, was determined as:

(4) $$s_{IC10}=\left(x_{H}-x_{L}\right)\times IC_{10}\sqrt{\frac{s_{V90}^{2}+s_{VL}^{2}}{{\left(V_{90}-V_{L}\right)}^{2}}+\frac{s_{VH}^{2}+s_{VL}^{2}}{{\left(V_{H}-V_{L}\right)}^{2}}-2\frac{s_{VL}^{2}}{\left(V_{H}-V_{L}\right)\times \left(V_{90}-V_{L}\right)}}$$

where $s_{IC10}$ is the standard error of *V*_90_, $s_{VL}$ is the standard error of *V_L_*, $s_{VH}$ is the standard error of *V_H_*.

For characterization of intracellular DOX and DOXol exposure there were two calculated parameters, cellular uptake ratio (*IC_UR_*) and intracellular concentration (*IC_C_*). These were calculated with Equations (5) and (6), and are based on the UPLC-MS measured concentration in the cell lysates.

In Equation (5), there are two main assumptions. Firstly, *IC_C_* is an average intracellular concentration that includes the higher concentration that is expected for the cell nucleus and mitochondria [2]. Secondly, it assumes that all cells are homogenous spheres with a diameter measured using a cell counter:

(5) $$IC_{C}=\frac{C_{lysate}\times v_{lysate}}{n_{cells}\times v_{cell}}$$

where $C_{lysate}$ is the lysate concentration in µM, $v_{lysate}$ is the lysate solution volume, $n_{cells}$ the number of cells, and $v_{cell}$ the volume per cell.

The cellular uptake ratio (*IC_UR_*) was calculated according to Equation (6). This is an estimation of the fraction of DOX that was taken up from the surrounding media irrespective of the concentration or formulation applied. This parameter corresponds to the cellular availability of the study drug and is as such a dimensionless parameter. In this parameter, the *IC_C_* for both DOX and its main metabolite DOXol was included, as DOX is metabolized to DOXol intracellularly:

(6) $$IC_{UR}=\frac{IC_{C\_DOX}+IC_{C\_DOXol}}{E_{Conc}}$$

where *E_Conc_* is the exposure concentration, *IC_C_DOX_* is the intracellular concentration of DOX and *IC_C_DOXol_* is the intracellular concentration of DOXol, the main metabolite of DOX (both *IC_C_DOX_* and *IC_C_DOX_*_o*l*_ were calculated using Equation (5)). All calculations were performed in Excel 2016 and supporting visual basic macros.

To judge the statistical significance of differences in responses for different cell lines, treatments and exposure times, mean values of IC_50_ and *IC_UR_* were directly compared to respective standard deviation as well as by applying two- and three-way ANOVA on the IC_50_ and *IC_UR_* data. For levels of factors containing more than two levels, the ANOVA was followed up by Tukey’s post hoc test. Since there were differences of one or several orders of magnitude between values (especially when comparing DOX_S_ and DOX_PL_ treatments), logarithmic values were used in these comparisons and tests, which also had the advantage of higher homoscedasticy of data. The statistical software Minitab was used in these analyses.

### 2.9. Physiologically Based Modeling

Clinical evaluation of the experimental results was performed applying a physiologically based modeling approach (see Supplementary Materials for details). A physiologically based pharmacokinetic (PBPK) model developed by Hanke et al. was adopted to describe the disposition of DOX [33]. Briefly, in this model the disposition was described by unspecific tissue distribution, specific binding to DNA, unspecific metabolic hepatic clearance, unspecific elimination to bile, glomerular filtration and enterohepatic re-cycling. Furthermore, the distribution was optimized so as to accommodate for distribution to DNA containing blood cells, cell membrane translocation distribution and informed by DNA binding constants. The parameters of metabolism, bile excretion, and distribution, which could not be adequately informed by prior knowledge, were estimated by simultaneously fitting the model to clinical plasma, blood, urine and feces reference data. For the purpose of this study, an adjustment to the reported model was made in terms of reducing (1/1000) the molecular radius of DOX. This was done in order to harmonize DOX translocation over the vascular endothelial with the original model and to achieve equivalent results when using the PBPK model structure for proteins and large molecules in PK-Sim.

A model to describe the disposition of DOX_PL_ was developed using the PBPK model structure for proteins and large molecules in PK-Sim [34]. DOX_PL_ was modeled as an entity eliminated by DOX and limited to vascular and interstitial distribution, in accordance with the functionality and size of the drug delivery system. Model development was initiated by establishment of DOX_PL_ distribution, where the overall distribution was described by the size of the liposome (40 nm radius assumed). The distribution was initially informed using reported information on initial (<24 h) liposomal distribution to plasma, liver, kidney, spleen and lung [35]. This signifies the liposomes distribution to the interstitial space as distribution to endosomes and intracellular space was not allowed. DOX_PL_ elimination, i.e., release of DOX, and further optimization of the distribution (fat, muscles, bone, heart, skin, intestines, pancreas) was subsequently estimated. The release of DOX was described under the assumption that this process predominately occurs by unspecific spontaneous disintegration of the liposomes. This is supported by comparable systemic disappearance of DOX_PL_ and unloaded liposomes [35,36]. In the model, this was parametrized as clearance via an unspecific enzyme homogenously distributed throughout the body. Optimization were performed using clinical reference data of measured total DOX (DOX and DOX_PL_) in plasma (see Supplementary Materials for more details on the model development and final model) [36].

A new parameter, cellular exposure concentration (C_Exp_), was established as a combination of the interstitial (C_Int_) and intracellular concentration for liver cells. This parameter symbolizes the total concentration that the cells of interest will be exposed to. The established models were subsequently used, together with measured in vitro *IC_UR_* ratios for DOX, to assess whether adequate DOX concentration levels in cancer cells are achieved, i.e., within target therapeutic zone, after a clinically relevant dose (50 mg/m^2^) DOX_S_ or DOX_PL_. The therapeutic zone was defined on the basis of the cell viability investigations as the concentration range above the lowest concentration with detectable effect (~10%).

## 3. Results

### 3.1. Cell Viability

Cell viability of HepG2, Huh7, SNU-449 and MCF-7 was measured after exposure to different concentrations of DOX_S_ and DOX_PL_ for 24, 48 and 72 h. This showed a difference in the sensitivity to both DOX_S_ and DOX_PL_ in the different human tumor cells, as shown by the (mean ± SD) IC_50_ values (µM) (Table S1 and Figures S1–S3 in Supplementary Materials). The IC_50_ values were more than one order of magnitude lower for the more sensitive cell lines HepG2, Huh-7 and MCF7, as compared to the more resistant cell line SNU449, under both DOX_S_ and DOX_PL_ treatment, which was statistically significant at all exposure times (*p* < 0.00005). In addition, Huh-7 showed a 3-fold difference to HepG2 and MCF7 when comparing Huh-7 with the other cell lines (*p* < 0.01). Furthermore, the relative difference between the mean IC_50_ values between HepG2 and MCF7 following both DOX_S_ and DOX_PL_ treatments showed no clear difference, despite their different tissue origins (*p* > 0.5 at any time point and treatment).

**Figure 1:**
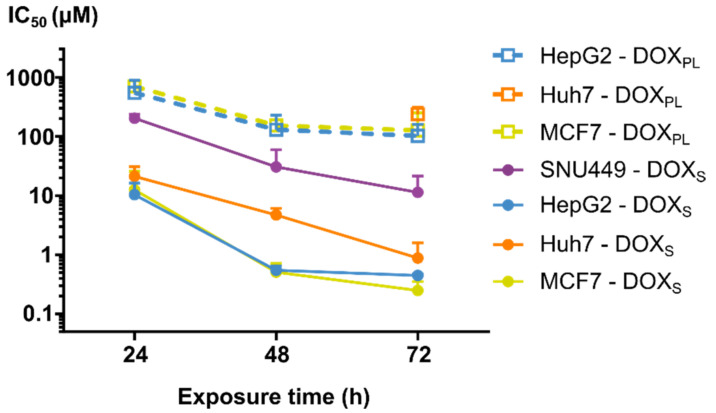
The mean (±SD) IC_50_ values for different treatments during the three exposure times (24 h, 48 h and 72 h). The anti-tumor responses in the different cell lines (SNU449 in purple, HepG2 in blue, Huh-7 in orange and MCF7 in yellow) using the two study formulations (hollow squares for DOX_PL_, circles for DOX_S_) are shown over the three different exposure times. The results for Huh-7 and SNU449 cells treated with DOX_PL_ were excluded (except for Huh-7 at 72 h, where only one out of three replicates was excluded), as the maximum dose was too low to calculate the IC_50_ values.

As expected, there was a clear trend that longer exposure times for DOX_S_ and DOX_PL_ reduced the IC_50_ values (Figure 1 and Table 1). From 24 to 48 h, the IC_50_ values showed a 4–18-fold decrease for all cell lines (*p* < 0.0005). From 48 to 72 h, the changes in IC_50_ values were considerably lower for HepG2, MCF7 and SNU449 cell lines, while the increasing trend seems to continue for Huh-7 under DOX_S_ treatment.

When comparing sensitivity to DOX_S_ versus DOX_PL_, it is notable that the IC_50_ values in all cancer cell models were higher for the DOX_PL_ than DOX_S_ within the same cell model. Specifically, in the HepG2 and MCF7, we observed approximately 10 times higher IC_50_ values for DOX_PL_ compared to DOX_S_, as they ranged between 760 ± 120 µM (24 h in MCF7) to 110 ± 62 µM (72 h in HepG2), respectively (Figure 1 and Table 1) (*p* < 0.00005). In the SNU449 cell line, and in some cases also for Huh7, the IC_50_ was not precisely determinable for DOX_PL_. However, where it was calculable, a similar relative difference between the treatments was observed as for the other cell lines.

**Figure 2:**
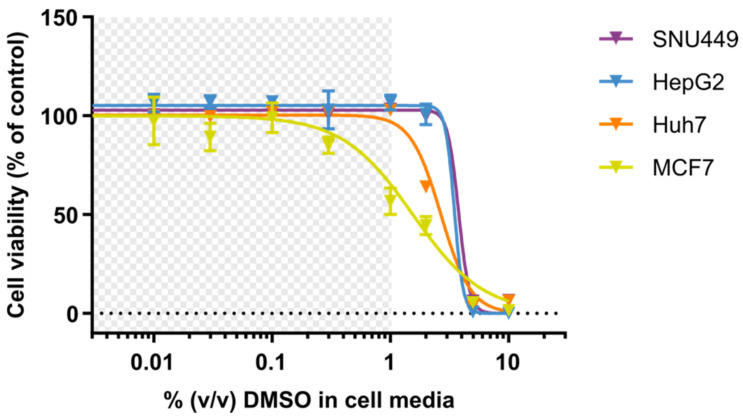
The mean (±SD) cell viability after 72 h treatment with the solvent DMSO in a concentration range of 0.01–10% DMSO in cell media. The different cell lines (SNU449 in purple, HepG2 in blue, Huh-7 in orange and MCF7 in yellow) were given CCM_Fed_ containing DMSO at different concentrations. The DMSO concentration range 0 to 1% DMSO (grey area) was applied during DOX_S_ treatment.

The cell viability of the DOX-treated human tumor cells was investigated after a 2 h pretreatment with lansoprazole (500 µM), and the IC_50_ data for DOX_S_ and DOX_PL_ are shown in Table 1. There were no clear trends in the effect of the PPI pretreatment on IC_50_ values for neither DOX_S_ nor DOX_PL_, and most of the pre-treatments had no appreciable effect. To assess whether the presence of DMSO in the DOX-solutions could have contributed to the toxicity, cell viability was determined after exposure to different concentrations of DMSO, as seen in Figure 2 (Table S1 in Supplementary Materials). The *IC*_10_ values for all PLC cell lines were above 1% DMSO, which was the highest concentration of DMSO given to the cells in the cell viability assay used to determine the IC_50_ values (1% DMSO at 1000 µM DOX_S_). This was not the case for MCF7, which had an IC_10_ of 0.23 ± 0.025% at 72 h exposure. While this is lower than the maximum dose of DMSO, the corresponding concentration of DOX (230 µM) was almost 10 times as much as was needed to kill all cells at 72 h exposure. These data suggest that DMSO was unlikely to contribute to toxicity of DOXs in any of the four different cell lines.

### 3.2. Intracellular Concentration and Uptake Ratio of DOX and DOXol Determined with a Bioanalytical Method Using UPLC-MS

In this study, a UPLC-MS method was developed to simultaneously quantify DOX and its primary metabolite DOXol in cell lysate (intracellular compartment) and in extracellular media. Matrix effects were most pronounced in the intracellular sample portions, suggesting ionization enhancement effects. Interestingly, extraction recoveries were higher for DOXol than DOX, but the isotopically labelled internal standards corrected for these differences (Table S2). The selectivity was demonstrated using matrix blanks injected before each calibration curve and sample. No peaks were detectable at the retention times corresponding to the analytes and internal standards of interest. After high-concentration calibrators or samples, blanks were injected and no carry-over peaks were observed. Quality control (QC) samples at low (0.1 µM), medium (1 µM) and high (10 µM) concentration levels, within the linear range, were prepared in triplicates for both analytes in each matrix and for each sample run. The relative bias and relative standard deviation for DOX QC samples were 3.0 and 11.7% (low), 5.4 and 10.4% (medium) and 0.5 and 6.0% (high), respectively. For DOXol QC samples, the numbers were −0.5 and 20.0% (low), 1.7 and 8.6% (medium), and −0.4 and 7.6% (high).

**Figure 3:**
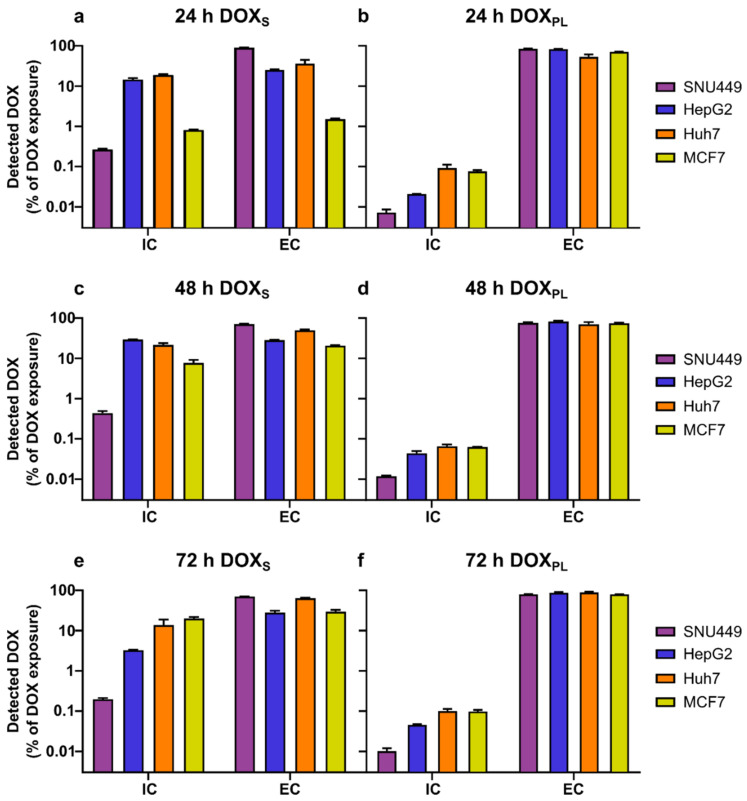
The mean (±SD) amount of DOX quantified inside (intracellular, IC) and outside (extracellular, EC) the cells, as a percentage of total added dose of DOX. (**a**,**c**,**e**) Cells treated with DOX_S_; (**b**,**d**,**f**) cells treated with DOX_PL_. The exposure concentration was based on the calculated IC_50_ of that treatment determined in this study, with a maximum concentration of 200 µM.

**Figure 4:**
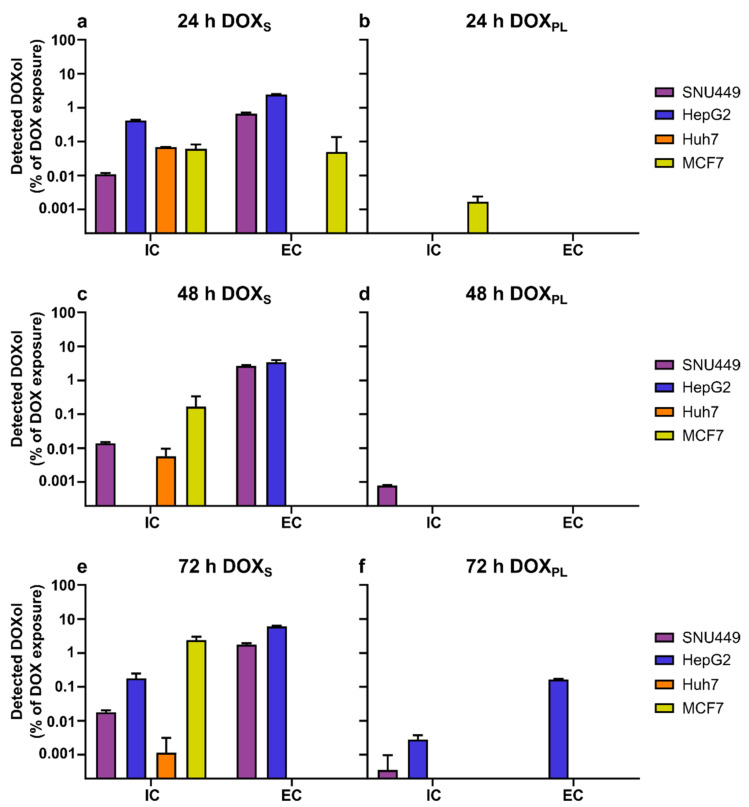
The mean (±SD) amount of DOXol quantified inside (intracellular, IC) and outside (extracellular, EC) the cells, as a percentage of total added dose of DOX. (**a**,**c**,**e**) Cells treated with DOX_S_; (**b**,**d**,**f**) cells treated with DOX_PL_. The exposure concentration was based on the calculated IC_50_ of that treatment determined in this study, with a maximum concentration of 200 µM.

In Table 2, the measured extra- and intracellular amounts of DOX and DOXol are shown. Cell lines were treated with extracellular concentrations of DOX_S_ or DOX_PL_ corresponding to their calculated IC_50_ values (Table 2). The maximum concentration of DOX_S_ or DOX_PL_ used was 200 µM. The relationship between intracellularly and extracellularly quantified DOX and DOXol is demonstrated in Figure 3 and Figure 4, respectively. The measured median diameter of the cells was 14 µm for SNU449, Huh7, MCF7, and 12 µm for HepG2. The number of tumor cells left after the end of the drug exposure period for each tested cell line is given in Table 3.

The intracellular uptake ratios (*IC_UR_*) for DOX_S_ and DOX_PL_ into the human cell lines during the three different treatment periods are given in Table 3. In all cell lines and treatment periods, the *IC_UR_* for DOX_S_ and DOX_PL_ ranged from 4.5 to 1500 and 0.12 to 5.2, respectively. In general, the ratio *IC_UR_* DOX_S_/*IC_UR_* DOX_PL_ had a median value of 87 across all cell lines and time points (*p* < 0.00005). The lowest *IC_UR_* and *IC_C_* of DOX for both formulations was observed for SNU449, which is in accordance with this cell line having the highest IC_50_-value (Table 1 and Table 3). The range of *IC_UR_* and intracellular concentration did not show any trend among these cell lines Huh7, MCF7, and HepG2.

DOXol was formed in all cell lines to a similar extent after 24 h exposure of DOX_S_ (Figure 4a). There was no difference between SNU449 and the three more sensitive cell lines, which suggests that metabolic formation of DOXol mediated by cytosolic enzymes, such as carbonyl reductases and/or aldo-keto reductases, does not explain the lower sensitivity of SNU449. The extracellular concentration of DOXol was higher at all exposure times for SNU449 and HepG2 when exposed for DOX_S_ which is most likely mediated by efflux proteins expressed in these cell lines. The efflux of DOXol was most pronounced in HepG2 and SNU449 (Figure 4a,c,e). In general, the formation of DOXol was far lower after treatment of DOX_PL_, which is in accordance with its lower *IC_UR_* of DOX as carbonyl reductases and/or aldo-keto reductases are located intracellularly (Figure 4b,d,f).

### 3.3. Physiologically Based Pharmacokinetic Modeling

**Figure 5:**
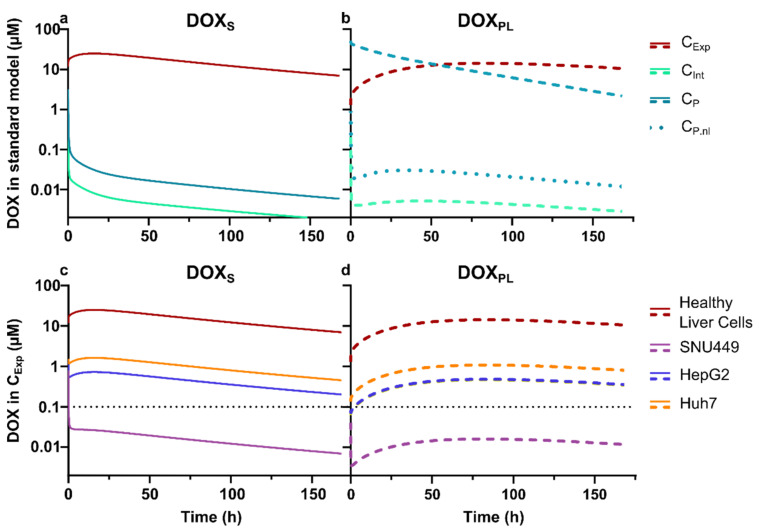
The results from the physiologically based simulation. (**a**,**b**) Simulated cellular exposure (C_Exp_, a combination of the interstitial and intracellular concentration for liver cells), interstitial concentration in the liver (C_Int_), and plasma concentration (C_P_) of DOX when given as DOX_PL_ and DOX_S_. Treatment was set to a dose of 50 mg/m^2^ for both treatments, with C_Exp_ in red, C_Int_ in green and plasma concentration in teal, for both DOX_S_ (full lines) and DOX_PL_ (dotted lines) as well as the non-liposomal DOX found in plasma after DOX_PL_ treatment (C_P.nl_, circles). (**c**,**d**) C_Exp_ is modelled based on experimental IC_UR_ for the PLC cells, with healthy liver cells (red), SNU449 (purple), HepG2 (blue) and Huh7 (orange). This is shown for DOX_S_ treatment (**a**,**c**) and DOX_PL_ treatment (**b**,**d**) at a dose of 50 mg/m^2^. The dotted line symbolizes the therapeutic zone, which was defined from the cell viability investigations as the concentration range above the lowest concentration with detectable effect (~10%). Simulations were performed with PBPK models for DOX_S_ and DOX_PL_, as described in Section 2.9, adopting a typical male individual (30 years old, 73 kg).

The model predicted extensive intracellular accumulation of DOX, mediated by the parametrization of DNA binding, where a maximum exposure to an interstitial DOX concentration ratio of ~10,000 was simulated for the liver (Figure 5a,b). Measured *IC_UR_* values for investigated cancer cell lines were adopted as a surrogate for intracellular accumulation in tumor tissue for different variants of HCC indicated possibilities for large differences in intracellular DOX exposure with potential consequences of susceptibility to DOX. The predicted average of 168 h cellular exposure, i.e., the sum of interstitial and intracellular concentration, was 0.5–1 µM for HepG2, Huh-7 and MCF7, while it was 40 times lower (0.02 µM) for SNU449 (Figure 5c). In relation to the in vitro viability investigation, this indicates that a SNU449-like tumor would not reach therapeutic intracellular DOX levels at a parenteral dose of 50 mg/m^2^. Similar levels, as well as differences, in cellular exposure between cell lines, were predicted for a simulation of DOX_PL_ (50 mg/m^2^) (Figure 5d). Notably, the total cellular exposure (AUC) was comparable between the two formulations (2500 and 1800 µM·h, respectively), despite a 500-fold higher plasma exposure after administered as DOX_PL_ compared to DOX_S_ (3.0 and 1600 µM·h, respectively).

## 4. Discussion

In this study, cytotoxic potency and its relation to intracellular exposure of DOX and its active metabolite doxorubicinol (DOXol) was investigated in four human cancer cell lines, following treatment of DOX as a solution (DOX_S_) or as the nano-sized pegylated liposome Doxil^®^ (DOX_PL_). Three human liver cancer cell lines (HepG2, Huh-7 and SNU449) and one human breast cancer cell line (MCF7), used as a reference, were selected based on previously published data and on their response to DOX [29]. The cell viability was determined by calculating IC_50_ values for DOX_S_ and DOX_PL_ at wide extracellular concentration ranges using a novel and more accurate approach, prioritizing the crucial concentrations in the mid part of the slope in the viability curves (Equations (2)–(4)). The effects on cell viability between the different cell lines and exposure times were related to the cellular uptake and intracellular concentrations of DOX and DOXol. This comparison was also extended into a theoretical model where extra- and intracellular concentration–time profiles of DOX were simulated to assess the significance of experimental *IC_UR_* values in terms of anti-tumor effect in a clinical situation. These findings drive the field forward in increasing the understanding how drug response between is affected by intracellular uptake of DOX, both in the context of preclinical research using cell lines, as well as the potential translation to the clinic through theoretical modelling.

HepG2 and MCF7 were the most sensitive cell lines to both treatments in this study [37,38]. HepG2 cells have been reported to have limited tumorigenic potential in in vivo xenograft models, and overall express less oncogenic proteins than other, more resistant, cell lines, such as Huh7 [29]. In this study, the Huh-7 cell line has been shown to be more resistant to DOX_PL_ than HepG2 and MCF7 at all-time points, while this difference is not as clear for DOX_S_. One potential explanation for the difference in cell viability is that HepG2 cells carry wild-type p53, while Huh-7 cells are characterized by a point-mutation in the p53-gene [39,40]. Inactivation of p53 has been shown to mediate resistance to DOX treatment in breast cancer cell lines and could thus play a similar role in determining sensitivity to DOX in the different PLC cell lines [39,41].

In this study, it was clearly demonstrated that SNU449 was the most resistant cell line against both treatments, which is in line with earlier reports [29,42]. A possible explanation for this increased resistance is the lower intracellular uptake ratio of DOX from both DOX_S_ and DOX_PL_ in the SNU449 cells, compared to the other cell lines. Membrane efflux, as well as other Fascin-1-mediated mechanisms that affect cell adhesion, cell–cell interactions and motility, could also be linked to the observed increased resistance [35]. The lower *IC_C_* could be a result of lower uptake and more extensive metabolism. An argument against the higher metabolism in this cell line is the results shown in Figure 4, where the lower intracellular uptake ratio in SNU449 did not correspond with a higher amount of DOXol formed, neither inside nor outside the cells, as compared to the more sensitive cell lines. Based on previous research, it is known that poorly differentiated cell lines, such as SNU449, provide better study models for human HCC profiles, and may contribute to understanding drug-resistance observed in clinical practice [43]. Our theoretical model simulations reveal that the level of resistance observed in SNU449 cells could lead to sub-therapeutic DOX exposure in a clinical context (50 mg/m^2^) (Figure 5c,d). This was irrespective of whether DOX was administered as a solution or as DOX_PL_, despite the large difference in total DOX plasma exposure, which is in accordance with DOX treatment being ineffective in some patients. Although a certain level of resistance was experimentally determined for the other PLC cell lines investigated (HepG2 and Huh-7), the cellular exposure was predicted to reach the therapeutic zone (>0.1 µM) after a 50 mg/m^2^ dose (Figure 5c,d). This further supports the importance of tumor characterization before selecting a strategy for therapeutic intervention, and emphasizes the potential of personalized medicine [44]. In addition, these data confirm SNU449 cells as a valuable study model for studying resistance to DOX and DOX_PL_ in HCC and for using multiple cell lines in cancer research, in order to draw conclusions that are relevant for different tumor phenotypes.

The lower IC_50_ values at longer exposure times observed in this study could be attributed to an overall increased cellular uptake of DOX and a subsequent accumulation inside the cell nucleus, where it induces cell death through different mechanisms, including ferroptosis [45,46]. However, the effect of further exposure time is significantly decreased or ceases between the 48 to 72 h time interval for the HepG2, SNU449 and MCF7 cell lines, while it might be maintained for Huh-7 [29,45,47,48]. The clinical benefit of an extended drug extracellular exposure time, which is a localized formulation strategy for TACE, can lead to a potential synergistic increase in intracellular uptake ratio of DOX and a prolonged intracellular retention time and effect duration. However, our results suggest that this synergistic effect might depend on the tumor phenotype and intercellular differences, as we did not observe this in all cell lines. The in vitro exposure times in our study were selected based on their clinical relevance for the local pharmacokinetics of DOX and DOXol following TACE injections with an emulsion-based formulation (Lipiodol^®^) [24]. The DOX plasma concentrations in HCC patients have been reported to be approximately in the range 0.2–30 µM for several days in central and peripheral veins, with corresponding DOXol plasma concentrations between 0.01–0.03 µM [14,49,50]. The total intracellular concentrations of DOX in the cell lines used in our study, when exposed to DOX_S_ or DOX_PL_ at their IC_50_ values, were several fold higher than their exposure concentrations (Table 3). With TACE, DOX is delivered directly to the hepatic tumor tissue, and the blood flow is then often embolized to maintain a high DOX concentration around the tumor, as well as to block the tumor’s blood supply [1,23]. In these cases, the local concentration in or around the tumor tissue in patients is expected to be substantially higher. Studies using VX2-rabbits have shown that total intra-tumoral DOX concentrations following conventional TACE of 2 mg/kg DOX were in the range 8–17 µM up to 3 days after dosing. In the same study, DOX was also given at the same dose in microspheres, with DOX intra-tumoral concentrations ranging between 20 to 65 µM [49]. In addition, multi-sampling of plasma in a domestic pig model (10–12 kg), showed that two repeated intravenous doses of 64 mg DOX resulted in resected liver tissue concentrations (corrected for blood contribution) of approximately 25 µM [50]. This concentration range corresponds with the PBPK model in this study (Figure 5).

There was a clear difference in cell viability between DOX_S_ or DOX_PL_ in all investigated cell lines, with DOX_S_ several-fold more potent than DOX_PL_. These ratios are in accordance with earlier reports, in which DOX_S_ was shown to be 10 to 1000 times as potent as DOX_PL_ during different exposure conditions [15,48]. Other in vitro studies have also reported that encapsulated DOX might have lower IC_50_ values than DOX_S_. For instance, Li et al. in 2020 reported DOX-loaded micelles in the range of 0.35–0.50 µM in three different cell lines (for HCT-116, HT-29 and SW480 cells) [51]. Their IC_50_ for free DOX was reported to be 2.6–3.1 µM [51]. These findings were also supported by comparing free DOX and DOX loaded in dendrimers, where IC_50_s were 1.4 µM and 0.50 µM, respectively [52]. In our study, we found that the *IC_C_* for DOX_PL_ was lower than for DOX_S_, showing a 1 to 3 log value difference for each cell line and time point. For MCF7, the IC_UR_ value increased with exposure time, while no clear trends can be noted in the other cell lines. A plausible hypothesis for the lower in vitro potency for DOX_PL_ is a low, variable and incomplete release of DOX extracellularly from the liposome (i.e., prior to DOX cellular uptake) [53]. Another explanation could also be a low cellular uptake of this particular nano-sized DOX_PL_ and/or low intracellular DOX release. Some reports propose that the main mode of action for DOX_PL_ is attributed to DOX that is released extracellularly and subsequently taken up by the cells [15,54]. There is currently no consensus regarding the extent of transport of the pegylated liposome (Doxil^®^) into cancer cell lines in vitro. Taking together our findings supporting a decreased potency of DOX_PL_ compared to DOX_S_ and the existing controversy in published literature, further research of the structure–activity relationships for cellular targeting and uptake for various types of TNP is encouraged, including pegylated liposomes.

DOX passes across cell membranes by passive lipoidal diffusion, as well as by carrier-mediated influx and efflux processes [3]. Cellular pharmacokinetics, cellular uptake and intracellular distribution have all been shown to be pH-dependent in accordance with the pH-partitioning model [10]. In this study, the extracellular pH ranged from 7.33–8.81 after treatment (Supplementary Material Table S3), which is expected to facilitate the passive membrane diffusion of DOX. In our in vitro study, the 2-h pretreatment of lansoprazole had no consistent effect on the observed IC_50_ values for DOXs or DOX_PL_ in the examined cell lines. One plausible explanation is that slightly acidic conditions (pH 6) are required in order to have a PPI-effect on DOX potency [55].

The high *IC_UR_* of DOX and the lack of distinct correlation with the extracellular concentration in the four cell lines suggests that passive diffusion is a key mechanism for the overall intracellular delivery of DOX into these cell lines, which is supported by previous studies [4,10]. The high intracellular concentration of DOX is possible due to the high binding and retention capacity of DOX to nuclear and mitochondrial DNA, which has been clearly reported to be main intracellular binding sites of DOX [26,56]. The high cellular accumulation of DOX after treatment of DOX_S_ might contribute to reduced anti-tumor effect in avascular tumor regions in patients, as it reduces the intercellular diffusion of deeper tumor regions.

The metabolite DOXol can be quantified in vivo to measure how efficiently DOX is taken up and metabolized by cells; however, this strategy is seldom replicated for in vitro cell assays [57]. This can possibly be related to failure in detecting the often low-abundant DOXol in various cellular compartments. Our approach to quantify DOXol in extra- and intracellular samples proved to be successful and revealed that the extracellular amount of DOXol exceeded the intracellular amount approximately two-fold, especially in HepG2 and SNU449, when exposed to DOX_S_. However, the concentration of DOXol was still overall higher intracellularly, as the liquid volume measured extracellularly was consistently higher compared to the intracellular volume. The difference in concentration might be due to the fact that DOXol was formed inside the cells and is still mostly bound to different intracellular compartments, reducing the diffusion and/or carrier-mediated transport of DOXol out of the cells. The quantification of DOX and DOXol is assumed to reflect one single compartment, which is a simplification as DOX most likely accumulates in the cell nucleus, as previously reported. Instead, this experimental setup gives an average intracellular concentration that encompasses both the “free” DOX in the cytoplasm as well as the DOX intercalated in the nuclear and mitochondrial DNA.

In all stages of drug discovery, there is a clear need to distinguish between the observed effects of the agent’s concentration-dependent potency and its potential active metabolites at the site of action. Improving the understanding of drug exposure at the site of action is especially relevant in pharmacological cellular or tissue models, as the fraction of the drug that reaches the site of action in the cell or some sub-cellular space is usually unknown. In this study, the IC_50_ values were calculated based on the determined cell viability data at various exposure concentrations using a novel and more accurate approach, which prioritizes crucial concentrations in the mid part of the slope in viability curves. As the cellular uptake is several folds higher for the sensitive cell lines (HepG2, MCF7 and Huh-7), the difference in the calculated in vitro IC_50_ could be attributed to cellular net uptake of DOX and intracellular disposition (such as metabolism, efflux and lysosometropic activity). These differences between cell lines, formulations and exposure time which affect the in vitro cellular uptake, intracellular retention and exposure, will affect cell viability after DOX-treatment and warrants further investigations in order to fully optimize the concept of localized tumor drug treatment.

## 5. Conclusions

Overall, the results of this study show DOX_S_ to be a more potent chemotherapeutic formulation compared to the nano-sized liposomal DOX_PL_ (Doxil^®^) during the applied in vitro conditions. This is most likely due to a slower and incomplete uptake of DOX from the liposomal formulation, which might be a result of lower extracellular availability of DOX when dosed as DOX_PL_. While preclinical studies have reported on the potential of TACE and TNPs combination therapy in HCC treatment, the data in this study demonstrate the need to design TNPs with a high drug load and a tumor triggered release of DOX [58]. We found that the total intracellular concentrations of DOX had a median value of 230 times higher than the exposure concentrations. This high concentration-independent cellular uptake and accumulation further supports the hypothesis that passive diffusion is a key transport mechanism for DOX in these cell lines. The potent anti-tumoral effect of DOX from both DOX_S_ and DOX_PL_ maintained over time (lower IC_50_), suggests that extending high local DOX concentration in the liver tumor during TACE treatment could be an efficient therapeutic strategy for HCC. The PBPK model used in this study provided a more accurate description of the DOX concentration–effect relationship. The performed simulations exemplify the relevance of in vitro to in vivo translations in the assessment of clinical consequence of experimental findings, and highlight the importance of tumor characterization before selecting a strategy for therapeutic intervention.

## Figures and Tables

**Table 1:** The calculated mean (±SD) IC50 values (µM) of the different cell lines exposed to DOXS or DOXPL for 24, 48 and 72 h with and without proton pump inhibitor (PPI) pretreatment.

SNU449				
	DOX_S_	DOX_S_ + PPI	DOX_PL_	DOX_PL_ + PPI
24 h	218 ± 38	405 ± 1.0	n/a	n/a
48 h	32.9 ± 31	79.5 ± 11	n/a	n/a
72 h	12.2 ± 11	6.10 ± 0.32	n/a	614 ± 75
Huh-7				
24 h	22.8 ± 10	15.2 ± 0.56	n/a	n/a
48 h	5.06 ± 1.5	5.89 ± 0.53	n/a	n/a
72 h	0.943 ± 0.78	2.15 ± 0.49	256 ± 82	504 ± 42
HepG2				
24 h	11.1 ± 6.4	4.13 ± 0.79	589 ± 370	225 ± 110
48 h	0.584 ± 0.11	0.428 ± 0.048	139 ± 108	122 ± 43
72 h	0.478 ± 0.031	0.531 ± 0.21	111 ± 62	26.4± 22
MCF7				
24 h	13.5 ± 14	1.56 ± 0.20	757 ± 150	292 ± 25
48 h	0.547 ± 0.22	0.409 ± 0.017	164 ± 79	571 ± 34
72 h	0.267 ± 0.11	0.119 ± 0.034	136 ± 140	60.3 ± 4.5

**Table 2:** The calculated amounts (mean± SD) intracellular DOX (IC_A_ DOX) and DOXol (IC_A_ DOXol), as well as the extracellular DOX (EC_A_ DOX) and DOXol (EC_A_ DOXol) of each exposure amount (E_A_). When the quantified amount was below the LLOQ, it is symbolized with “n/a”. Each E_A_ was based on the calculated IC_50_ of that treatment with a maximum E_A_ of 3000 nmol. Please note that IC_A_ DOXol is reported in pmol, while the other amounts are reported in nmol.

SNU449						
Exposure Time	Treatment	E_A_ (nmol)	IC_A_ DOX (nmol)	IC_A_ DOXol (pmol)	EC_A_ DOX (nmol)	EC_A_ DOXol (nmol)
24 h	DOX_S_	3000	7.94 ± 0.44	332 ± 25	2700 ± 13	20.3 ± 1.3
	DOX_PL_	3000	0.218 ± 0.040	n/a	2540 ± 49	n/a
48 h	DOX_S_	450	1.96 ± 0.25	63.0 ± 48	321 ± 6.8	12.0 ± 0.71
	DOX_PL_	3000	0.352 ± 0.012	23.8 ± 0.80	2280 ± 86	n/a
72 h	DOX_S_	150	0.297 ± 0.021	25.8 ± 3.5	106 ± 1.1	2.54 ± 0.25
	DOX_PL_	3000	0.295 ± 0.0526	10.3 ± 18	2310 ± 39	n/a
**HepG2**						
24 h	DOX_S_	150	21.8 ± 1.9	631 ± 40	37.9 ± 1.2	3.67 ± 0.15
	DOX_PL_	3000	0.624 ± 0.0092	n/a	2490 ± 46	n/a
48 h	DOX_S_	7.5	0.293 ± 0.017	n/a	2.12 ± 0.034	0.256 ± 0.043
	DOX_PL_	1500	0.659 ± 0.087	n/a	1230 ± 58	n/a
72 h	DOX_S_	7.5	0.245 ± 0.0060	12.9 ± 4.9	2.10 ± 0.19	0.435 ± 0.023
	DOX_PL_	1500	0.663 ± 0.023	40.4 ± 14	1260 ± 63	2.39 ± 0.083
**Huh-7**						
24 h	DOX_S_	300	56.3 ± 3.1	210 ± 1.1	108 ± 25	n/a
	DOX_PL_	3000	2.76 ± 0.58	n/a	1600 ± 240	n/a
48 h	DOX_S_	75	16.2 ± 1.8	4.33 ± 2.9	37.3± 1.9	n/a
	DOX_PL_	3000	1.96 ± 0.21	n/a	2110 ± 270	n/a
72 h	DOX_S_	15	2.06 ± 0.77	n/a	9.59 ± 0.12	n/a
	DOX_PL_	3000	2.92 ± 0.36	13.3 ± 1.2	2600 ± 92	n/a
**MCF7**						
24 h	DOX_S_	150	1.22 ± 0.035	92.5 ± 33	2.26 ± 0.087	0.0758 ± 0.13
	DOX_PL_	3000	2.28 ± 0.19	50.8 ± 22	2140 ± 35	n/a
48 h	DOX_S_	7.5	0.579 ± 0.11	12.5 ± 13	1.51 ± 0.035	n/a
	DOX_PL_	3000	1.89 ± 0.014	n/a	2240 ± 84	n/a
72 h	DOX_S_	4.5	0.901 ± 0.076	104 ± 27	1.29 ± 0.13	n/a
	DOX_PL_	1500	1.41 ± 0.13	n/a	1150 ± 15	n/a

**Table 3:** The number of cells that were quantified, calculated mean (±SD) intracellular concentration (IC_C_) of DOX and DOXol and intracellular uptake ratio (IC_UR_) for DOX in the different cell lines at different exposure concentrations (E_Conc_). When the quantified amount was below the LLOQ, it is symbolized with “n/a”. Each E_Conc_ was based on the calculated IC_50_ of that treatment with a maximum E_Conc_ of 200 µM, see Table 1 for more details.

SNU449						
Exposure Time	Treatment	E_Conc_ (µM)	Cells after Treatment (M)	IC_C_ DOX (µM)	IC_C_ DOXol (µM)	IC_UR_
24 h	DOX_S_	200	2.77 ± 0.044	2000 ± 140	83.4 ± 7.0	10.4 ± 0.73
	DOX_PL_	200	3.37 ± 0.86	47.8 ± 18	n/a	0.24 ± 0.088
48 h	DOX_S_	30	3.29 ± 0.044	414 ± 48	13.3 ± 0.87	14.3 ± 1.6
	DOX_PL_	200	6.07 ± 0.26	40.5 ± 3.1	2.73 ± 0.031	0.22 ± 0.016
72 h	DOX_S_	10	5.04 ± 0.43	41.1 ± 3.6	3.59 ± 0.75	4.47 ± 0.38
	DOX_PL_	200	8.86 ± 0.54	23.2 ± 4.2	0.81 ± 1.4	0.12 ± 0.028
**HepG2**						
24 h	DOX_S_	10	6.21 ± 0.62	5090 ± 680	147 ± 20.6	523 ± 70
	DOX_PL_	200	5.71 ± 2.5	178 ± 76	n/a	0.89 ± 0.38
48 h	DOX_S_	0.5	4.87 ± 0.67	87.1 ± 8.1	n/a	174 ± 16
	DOX_PL_	100	5.89 ± 0.17	161 ± 21	0.77 ± 1.28	1.61 ± 0.212
72 h	DOX_S_	0.5	3.26 ± 0.30	108 ± 7.9	5.62 ± 1.7	228 ± 15
	DOX_PL_	100	4.17 ± 0.19	228 ± 17	14.0± 4.9	2.42 ± 0.18
**Huh-7**						
24 h	DOX_S_	20	6.32 ± 0.35	6 220 ± 590	23.2 ± 1.3	312 ± 29
	DOX_PL_	200	4.72 ± 0.35	412 ± 120	0.27 ± 0.47	2.1 ± 0.58
48 h	DOX_S_	5	3.01 ± 2.1	7 450 ± 8 400	2.34 ± 2.9	1490 ± 1 700
	DOX_PL_	200	6.06 ± 1.6	233 ± 43	n/a	1.16 ± 0.21
72 h	DOX_S_	1	5.28 ± 0.55	280 ± 125	0.024 ± 0.042	280 ± 120
	DOX_PL_	200	6.46 ± 0.39	314 ± 38	0.14 ± 0.16	1.57 ± 0.19
**MCF7**						
24 h	DOX_S_	10	5.65 ± 0.82	151 ± 21	11.2 ± 2.5	16.2 ± 1.9
	DOX_PL_	200	5.55 ± 0.80	292 ± 60	6.67 ± 3.4	1.49 ± 0.31
48 h	DOX_S_	0.5	3.51 ± 0.16	115 ± 17	2.56 ± 2.7	234 ± 33
	DOX_PL_	200	2.34 ± 0.61	593 ± 180	0.10 ± 0.10	2.96 ± 0.89
72 h	DOX_S_	0.3	3.69 ± 0.51	174 ± 41	19.5 ± 2.8	644 ± 130
	DOX_PL_	100	1.93 ± 0.33	517 ± 87	n/a	5.17 ± 0.87

## Supplementary Materials

The following are available online at https://www.mdpi.com/article/10.3390/cells10071717/s1, Figure S1: 24 h exposure to DOX_S_/DOX_PL_, Figure S2: 48 h exposure to DOX_S_/DOX_PL_, Figure S3: 72 h exposure to DOXS/DOXPL, Table S1: The calculated mean (± SD) IC_10_-values of the different cell lines exposed to DMSO for 24, 48 and 72 h, Table S2: Matrix effects and extraction recoveries, Table S3: Exposure media average pH, Supplementary material: PBPK model development.
