# Supplementary material for: In Vitro Cell Toxicity and Intracellular Uptake of Doxorubicin Exposed as a Solution or Liposomes: Implications for Treatment of Hepatocellular Carcinoma
Source: Cells. 2021 Jul 6;10(7):1717. doi: 10.3390/cells10071717 (PMC8306283; doi:10.3390/cells10071717)
Supplement: Supplementary file 1 [file cells-10-01717-s001.zip › cells-1229029-supplementary.pdf]

Figure S1. 24 h exposure to DOX_S/DOX_{PL}.

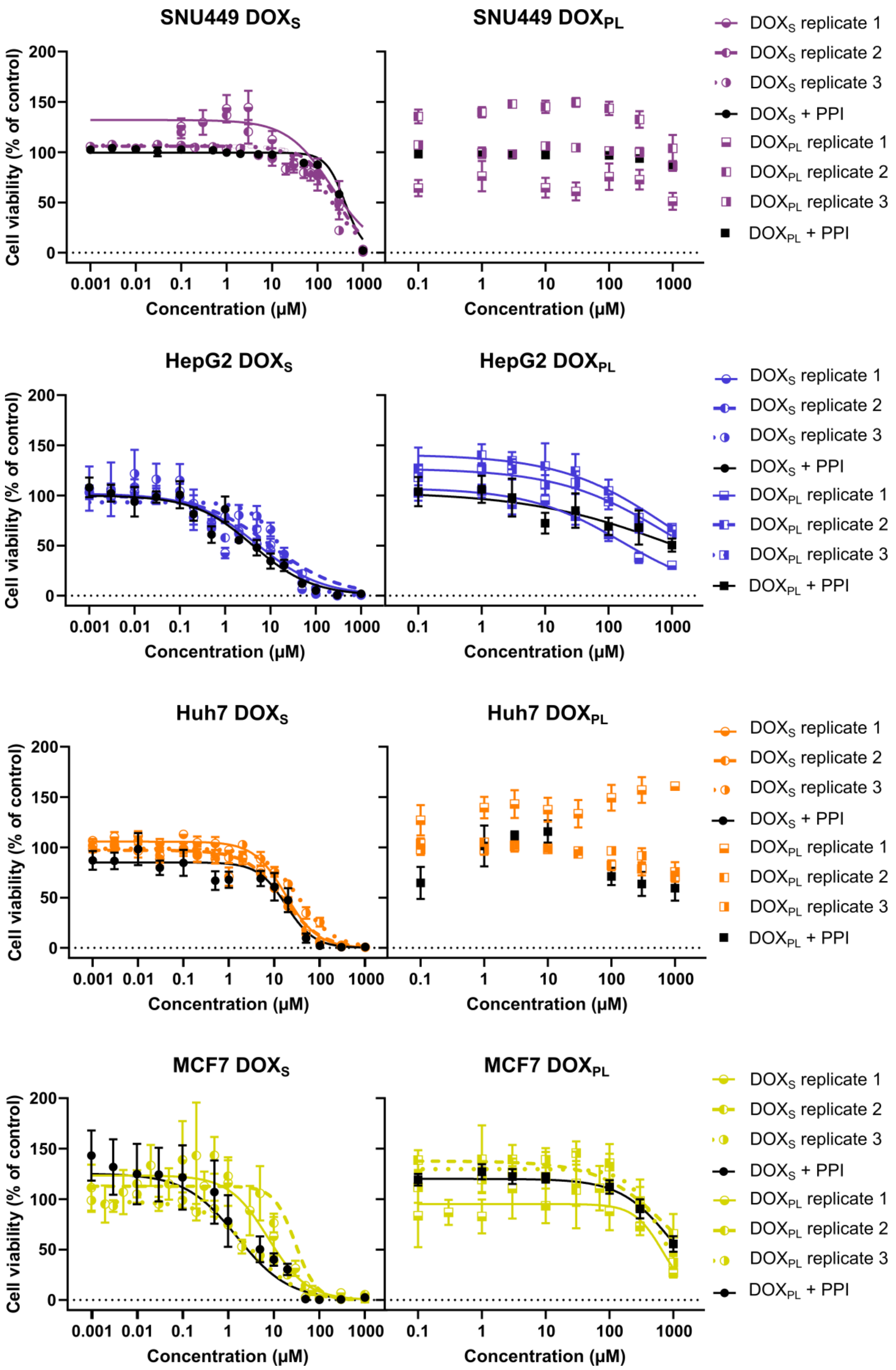

Figure S2. 48 h exposure to DOX_S/DOX_{PL}.

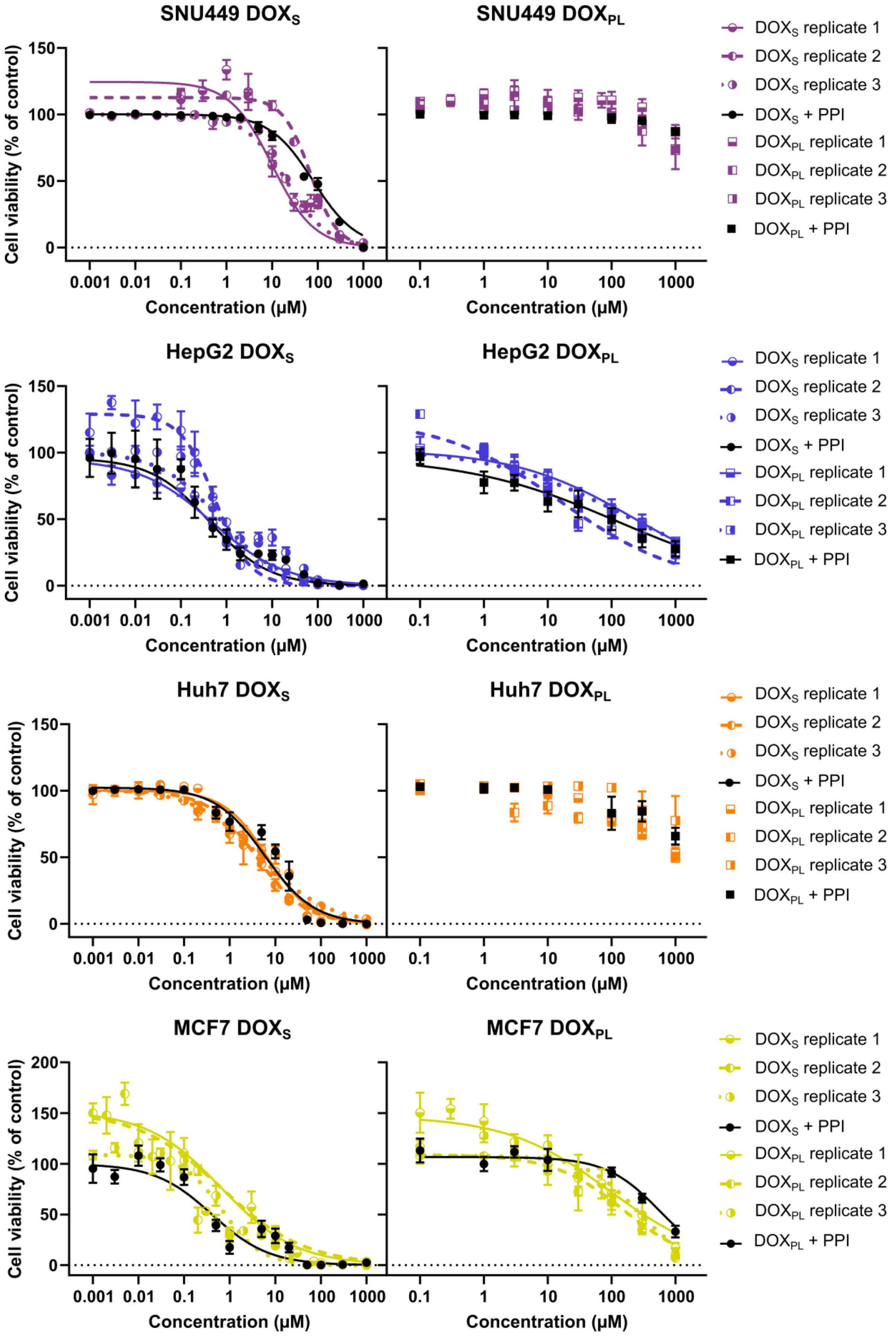

Figure S3. 72 h exposure to DOX_S/DOX_{PL}.

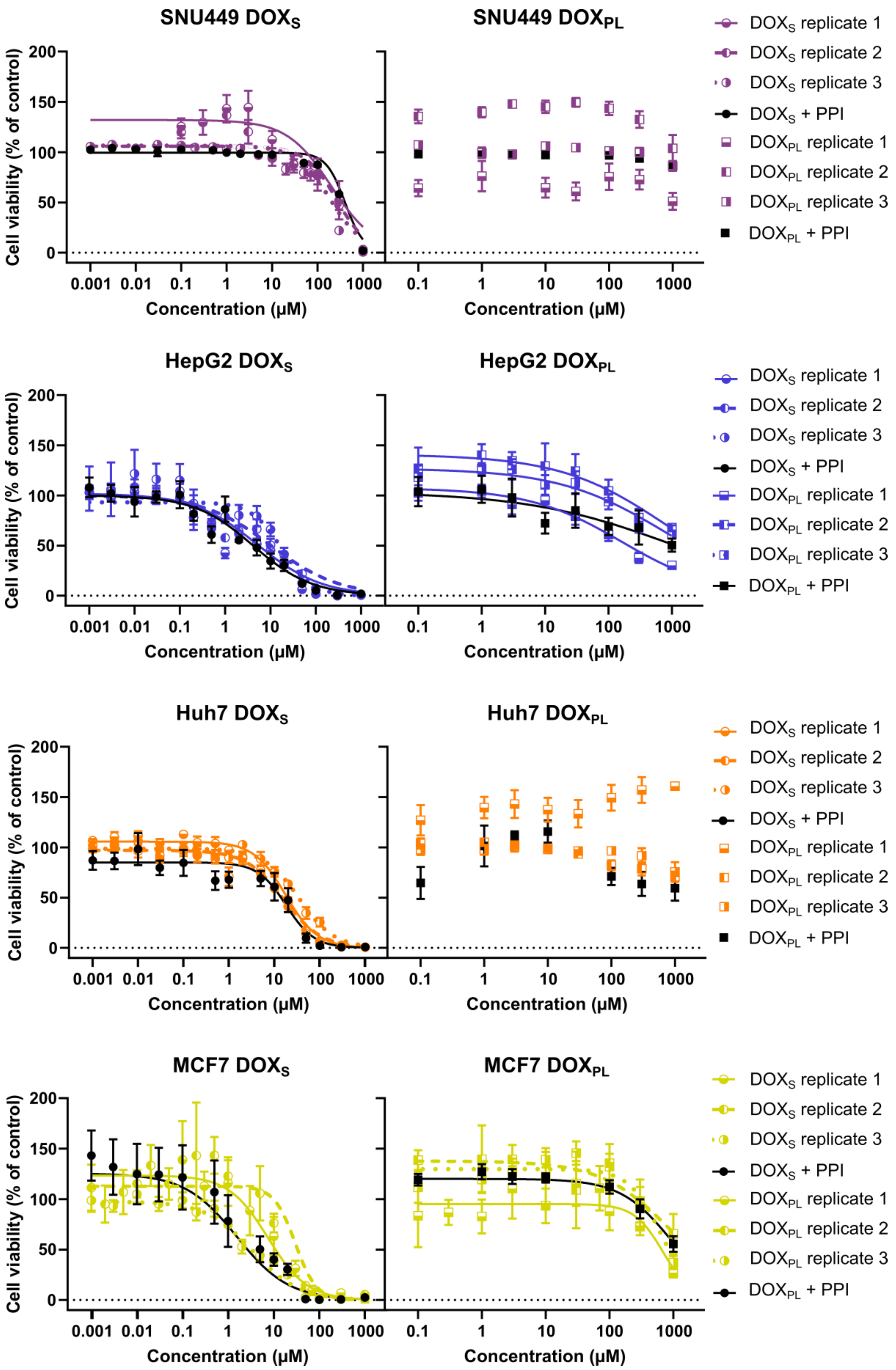

Table S1. The calculated mean (± SD) IC_{10}-values of the different cell lines exposed to DMSO for 24, 48 and 72 hours (% DMSO in the cell media)

Exposure time	SNU449	HepG2	Huh7	MCF7
24 h	2.72 ± 0.33	1.13 ± 0.11	1.24 ± 0.13	0.768 ± 0.40
48 h	2.08 ± 0.066		1.26 ± 0.038	0.258 ± 0.048
72 h	2.28 ± 0.025	2.19 ± 0.037	1.30 ± 0.019	0.229 ± 0.025

Table S2. Matrix effects and extraction recoveries (%) were evaluated using collected matrix components (portions) from the MCF7 cell line at two spiked concentration levels according to Matuszewski et al. 2003. Note that DOX from DOX_{PL} means that DOX_{PL} was spiked and DOX was quantified.

**MCF-7**	**Analytes**	**Matrix Effects**		**Recovery**	
		**25 µM**	**100 µM**	**25 µM**	**100 µM**
**Intracellular portion**	DOX	114%	103%	52%	58%
	DOX from DOX_{PL}	111%	102%	56%	59%
	DOX IS	112%	101%	54%	60%
	DOXol	111%	102%	72%	77%
	DOXol IS	112%	103%	72%	78%
**Exposure media portion**	DOX	92%	94%	88%	89%
	DOX from DOX_{PL}	89%	95%	89%	87%
	DOX IS	91%	92%	89%	89%
	DOXol	86%	91%	98%	97%
	DOXol IS	86%	92%	99%	97%
**Supernatant portion**	DOX	97%	96%	94%	93%
	DOX from DOX_{PL}	95%	95%	90%	92%
	DOX IS	97%	94%	94%	93%
	DOXol	92%	94%	100%	99%
	DOXol IS	94%	95%	100%	97%
**PBS wash portion**	DOX	80%	94%	121%	110%
	DOX from DOX_{PL}	89%	94%	109%	105%
	DOX IS	79%	93%	122%	110%
	DOXol	74%	91%	123%	111%
	DOXol IS	74%	91%	123%	110%

Table S3. Exposure media average pH (± SD) was measured after treatment (n=3). Samples without SD are n=1 due to limited sample volumes. HepG2 control samples were not measured due to not enough sample remaining after UPLC-MS analysis.

**Cell line**	**Treatment**	**Time (h)**	**Exposure concentration (µM)**	**Exposure media pH**
HepG2	DOX_S	24	10	8.49 ± 0.05
HepG2	DOX_S	48	0.5	8.45 ± 0.09
HepG2	DOX_S	72	0.5	8.45 ± 0.04
HepG2	DOX_{PL}	24	200	8.81 ± 0.02
HepG2	DOX_{PL}	48	100	8.76 ± 0.04
HepG2	DOX_{PL}	72	100	8.68 ± 0.02
Huh7	Control	24	0	8.43
Huh7	Control	48	0	8.17 ± 0.04
Huh7	Control	72	0	8.15 ± 0.03
Huh7	DOX_S	24	20	8.64 ± 0.07
Huh7	DOX_S	48	5	8.29 ± 0.07
Huh7	DOX_S	72	1	7.96 ± 0.06
Huh7	DOX_{PL}	24	200	8.56 ± 0.04
Huh7	DOX_{PL}	48	200	8.35 ± 0.10
Huh7	DOX_{PL}	72	200	8.20 ± 0.05
MCF7	Control	24	0	8.66 ± 0.04
MCF7	Control	48	0	8.66 ± 0.05
MCF7	Control	72	0	8.68 ± 0.01
MCF7	DOX_S	24	10	8.42 ± 0.06
MCF7	DOX_S	48	0.5	8.48 ± 0.13
MCF7	DOX_S	72	0.3	8.60 ± 0.21
MCF7	DOX_{PL}	24	200	8.08 ± 0.19
MCF7	DOX_{PL}	48	200	8.25 ± 0.04
MCF7	DOX_{PL}	72	100	8.29 ± 0.03
SNU449	Control	24	0	8.51
SNU449	Control	48	0	7.83 ± 0.09
SNU449	Control	72	0	7.78± 0.02
SNU449	DOX_S	24	200	8.41 ± 0.03
SNU449	DOX_S	48	30	7.94 ± 0.03
SNU449	DOX_S	72	10	7.56 ± 0.03
SNU449	DOX_{PL}	24	200	8.20 ± 0.05
SNU449	DOX_{PL}	48	200	7.64 ± 0.08
SNU449	DOX_{PL}	72	200	7.33 ± 0.02

Supplementary material: PBPK model development

This section of the supplementary material describes the model development of the PBPK models applied in the study. The aim of modeling in the current study was to visualize potential clinical implications of different level of cellular distribution, i.e., cancer resistance. The purpose was hence to facilitate rank order translation of the experimental cell assay measurements into a clinical perspective. We do not claim that the proposed model is fully validated, as such work requires more reference data and potentially additional sophistication to model structure and parametrization, and such investigation was outside the scope of this study.

For final models, e.g., parameter values and settings, the authors refer to the supplementary model file “Supplementary Material – PBPK models”

Doxorubicin model:

Setup of model:

The model presented by Hanke et al 2018 was adopted to describe doxorubicin (DOX). This model was developed using the “small molecule” structure in PK-Sim. In this study we wanted to use the “large molecule” structure to describe the disposition of the liposomes. Introducing a resistance for capillary distribution, by the two-pore theory, influenced the output for DOX. Equivalent results for DOX when using the “large molecule” model as with the “small molecule” model was achieved by reducing the molecular radius of DOX a 1000-fold. In the model this parameter is only used to calculate the resistance for capillary translocation.

Performance of Doxorubicin model can be seen in Figure A4 below:

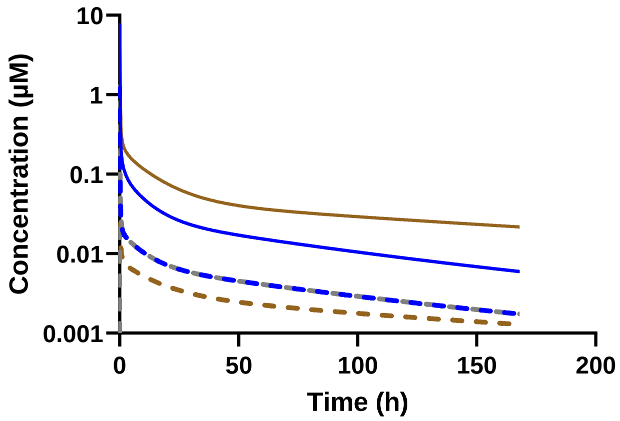

Figure S4. Simulated doxorubicin concentration time profiles in plasma (solid) and liver interstitial compartment (dashed). Color represents output from the original model presented by Hanke et al. (grey), adopting the “large molecule” functionality with same input parameters as presented by Hanke et al. (brown) and the model used in this study (blue) i.e., adopting the “large molecule” functionality with a reduction in doxorubicin molecule size but in other respect the same input parameters as presented by Hanke et al. The grey and blue lines are superimposed.

Liposome/Doxil model:

Data from Harrington et al 2001 and Gabizon et al. 1994 was used for liposome model development.

1. Distribution data of ^{111}In-DTPA pegylated liposomes in Harrington et al 2001.

Normal organ uptake of ^{111}In-DTPA pegylated liposomes as determined in plasma and by regions of interest (liver, spleen, lung and kidney) on the whole body gamma camera scans given for whole organ uptake (%), assuming correction of vascular distribution, was extracted from the report. Data up to 24h was used to reduce influence of liposomal disruption and the data was normalized to % activity at t=0.5 h.

2. As the plasma concentrations of total and encapsulated DOX reported in Gabizon et al 1994 are indistinguishable, i.e., negligible free DOX in the blood stream compared to encapsulated DOX, this enables using encapsulated DOX data for modeling of liposome disposition. There is an uncertainty of the fate of the liposomes but for this modeling exercise it was assumed that the "elimination" process releases DOX to the system as the liposomes spontaneously "dissolves".

Setup of model:

a) Doxil is defined as a compound (building block in PK-Sim).

b) Doxil Mw was set to the molecular weight of DOX (543.525 g/mol) to be able to relate model output to reported encapsulated DOX plasma concentration data as reference given an administered dose DOX.

c) The disposition of encapsulated DOX = DoxilDOX, assumes

    a. Size = 40 nm → can only distribute to vascular and interstitial space.

    b. Doxils ability to enter the interstitial space was adjusted ("optimized") by optimization of the "permeability through large pores" using the initial timepoints from the tissue distribution of empty Doxil reported in Harrington and Gabizon.

    c. No distribution to endosomes was allowed.

    d. Elimination of Doxil, i.e., also the release of DOX, was modelled to occur via two first-order processes (Fast + Slow).

d) The release was parameterized in PK-Sim as enzymatic processes where the release-mediating-enzyme was expressed in all compartments at the same concentration (=same release capacity in all compartments).

e) Specification of dedicated slow and fast release process was enabled by independently creating compound building blocks for Slow and Fast release. To achieve identification the fraction of dose released by the Slow and Fast process was set to 90% and 10%, respectively. The total release was identified using clinical data from Gabizon 1994 on plasma concentrations of total DOX as reference.

The parameter optimization of release rate constants and tissue distribution (permeability through large pores) was performed using the Monte-Carlo algorithm available in the "Parameter Identification" functionality within PK-Sim. Briefly, this is a statistical optimization functionality where minimization of the residuals between observed data and corresponding simulation output is done by varying selected input parameters in a given range of parameter values.

Performance of Doxil model:

The final model was able to describe the liposomal disposition sufficiently well in terms of distribution and elimination (Figure A5 and Figure A6).

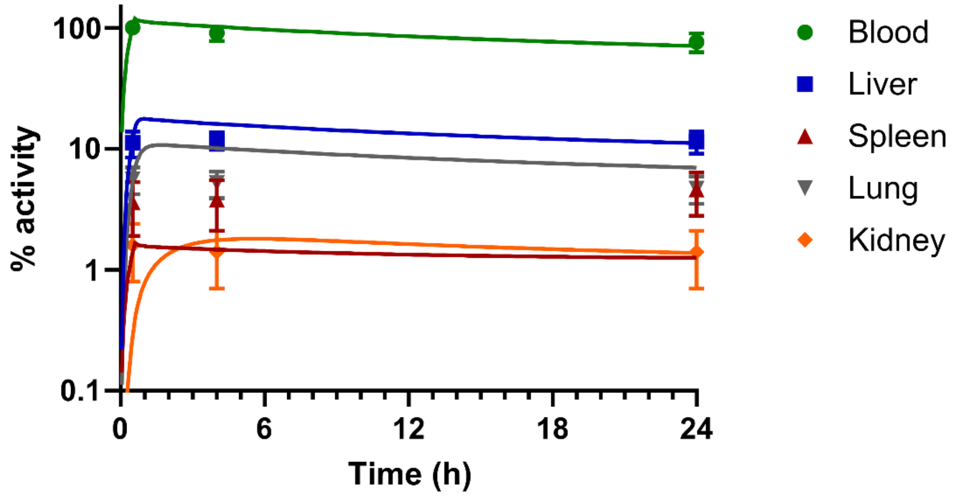

Figure S5. Observed (symbols) and simulated (lines) distribution of pegylated liposomes in plasma (green) and regions of interest (liver [blue], spleen [red], lung [grey] and kidney [orange]) displayed in log-linear scale. Observations (mean ± SD) were extracted from Harrington et al 2001. Simulated data was attained with Doxil PBPK model.

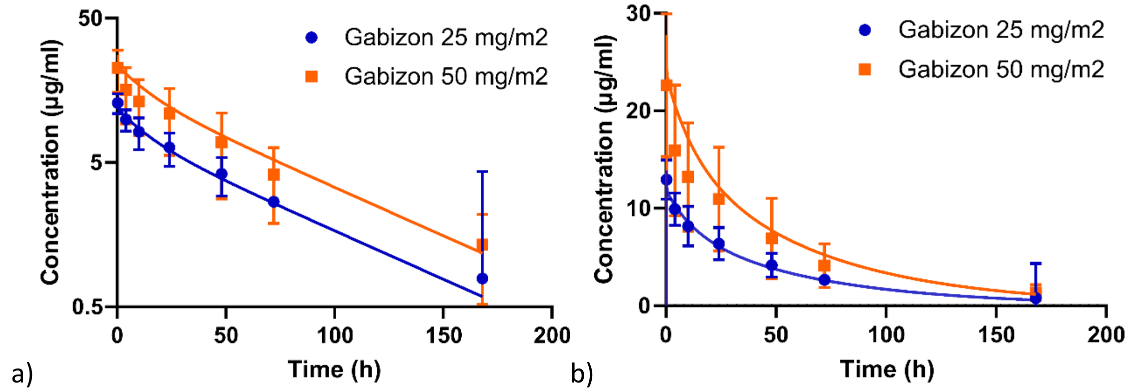

Figure S6. Observed (symbols) and simulated (lines) plasma concentration of encapsulated DOX in Doxil after administration of a total dose of DOX equal to 50 mg/m^2 (orange) and 25 mg/m^2 (blue) displayed in a) linear and b) log-linear scale. Observations (mean ± SD) were extracted from Gabizon et al. 1994. Simulated data was attained with Doxil PBPK model.

References:

Hanke N, Teifel M, Moj D, Wojtyniak JG, Britz H, Aicher B, Sindermann H, Ammer N, Lehr T. Cancer Chemother Pharmacol. 2018 Feb;81(2):291-304. A physiologically based pharmacokinetic (PBPK) parent-metabolite model of the chemotherapeutic zoptarelin doxorubicin-integration of in vitro results, Phase I and Phase II data and model application for drug-drug interaction potential analysis.

Harrington KJ, Mohammadtaghi S, Uster PS, Glass D, Peters AM, Vile RG, Stewart JS. Clin Cancer Res. 2001 Feb;7(2):243-54. Effective targeting of solid tumors in patients with locally advanced cancers by radiolabeled pegylated liposomes.

Gabizon A, Catane R, Uziely B, Kaufman B, Safra T, Cohen R, Martin F, Huang A, Barenholz Y. Cancer Res. 1994 Feb 15;54(4):987-92. Prolonged circulation time and enhanced accumulation in malignant exudates of doxorubicin encapsulated in polyethylene-glycol coated liposomes.
